# Film-based resources for grief management in medical education

**DOI:** 10.1192/j.eurpsy.2022.1591

**Published:** 2022-09-01

**Authors:** S. Tei, J. Fujino

**Affiliations:** 1 Kyoto University, Department Of Psychiatry, kyoto, Japan; 2 Waseda University, Institute Of Applied Brain Sciences, Saitama, Japan; 3 Showa University, Medical Institute Of Developmental Disabilities Research, Tokyo, Japan; 4 Tokyo International University, School Of Human And Social Sciences, Saitama, Japan; 5 Tokyo Medical and Dental University, Department Of Psychiatry And Behavioral Sciences, Graduate School Of Medical And Dental Sciences, Tokyo, Japan

**Keywords:** grief, bereavement, film, sense of coherence

## Abstract

**Introduction:**

Grief and bereavement are commonplace in clinical practice but have become a more pervasive issue because of the coronavirus 2019 pandemic. Consequently, the need for investigations, learning, and education about complicated grief and prolonged grief have been highlighted. Meanwhile, film-based teaching resources concerning grief care have been employed to complement curricula in medical education.

**Objectives:**

To explore how the grieving experience can be better communicated and mitigated, and explain how a film-based resource can be applied to improve the understanding of this issue.

**Methods:**

We reviewed and analyzed the meaning and cause of complicated, prolonged, disenfranchised grief, as well as related experiences (e.g., survivor guilt) featured in selected films. We discussed the interpretation of these films with medical students and faculty, based on a previously described approach [1].

**Results:**

We recaptured the roles of empathic communications and resilience skills in grief care. They bring a sense of coherence (SOC) or meaning to life by prompting the sharing of grief experiences, helping to reconstruct and contextualize a person’s loss, and assuaging feelings of worthlessness and hopelessness. Incidentally, recent studies have suggested that complicated and prolonged grief involves alterations in brain functioning of the reward system.

**Conclusions:**

This film-based approach utilizes vicarious experiences to better understand grief management. It allows the learner to more easily recognize that SOC, flexible situation-adjusted empathy, and the sharing of resources for improved communication to promote self-care are essential for patients, their families, as well as psychiatrists themselves. [1] Sondheimer, A. The life stories of children and adolescents. *Acad Psychiatry.* 2000:24(4):214–24.

**Disclosure:**

No significant relationships.

